# Sertraline, Paroxetine, and Chlorpromazine Are Rapidly Acting Anthelmintic Drugs Capable of Clinical Repurposing

**DOI:** 10.1038/s41598-017-18457-w

**Published:** 2018-01-17

**Authors:** Janis C. Weeks, William M. Roberts, Caitlyn Leasure, Brian M. Suzuki, Kristin J. Robinson, Heather Currey, Phurpa Wangchuk, Ramon M. Eichenberger, Aleen D. Saxton, Thomas D. Bird, Brian C. Kraemer, Alex Loukas, John M. Hawdon, Conor R. Caffrey, Nicole F. Liachko

**Affiliations:** 1 Institute of Neuroscience, University of Oregon, Eugene, OR 97403 USA; 2 Department of Microbiology, Immunology and Tropical Medicine, George Washington University, Washington D.C., 20052 USA; 3 Center for Discovery and Innovation in Parasitic Diseases, Skaggs School of Pharmacy and Pharmaceutical Sciences, University of California San Diego, 9500 Gilman Drive, La Jolla, CA 92093 USA; 4 Geriatrics Research Education and Clinical Center, Veterans Affairs Puget Sound Health Care System, Seattle, WA 98108 USA; 5 Centre for Biodiscovery and Molecular Development of Therapeutics, Australian Institute of Tropical Health and Medicine, James Cook University, Cairns, QLD 4878 Australia; 6 Department of Neurology, University of Washington, Seattle, Washington, 98195 USA; 7 Department of Psychiatry and Behavioral Sciences, University of Washington, Seattle, Washington, 98195 USA; 8 Department of Medicine, Division of Medical Genetics, University of Washington, Seattle, WA 98104 USA; 9 Department of Medicine, Division of Gerontology and Geriatric Medicine, University of Washington, Seattle, WA 98104 USA; 10 Department of Pathology, University of Washington, Seattle, Washington, 98195 USA

## Abstract

Parasitic helminths infect over 1 billion people worldwide, while current treatments rely on a limited arsenal of drugs. To expedite drug discovery, we screened a small-molecule library of compounds with histories of use in human clinical trials for anthelmintic activity against the soil nematode *Caenorhabditis elegans*. From this screen, we found that the neuromodulatory drugs sertraline, paroxetine, and chlorpromazine kill *C. elegans* at multiple life stages including embryos, developing larvae and gravid adults. These drugs act rapidly to inhibit *C. elegans* feeding within minutes of exposure. Sertraline, paroxetine, and chlorpromazine also decrease motility of adult *Trichuris muris* whipworms, prevent hatching and development of *Ancylostoma caninum* hookworms and kill *Schistosoma mansoni* flatworms, three widely divergent parasitic helminth species. *C. elegans* mutants with resistance to known anthelmintic drugs such as ivermectin are equally or more susceptible to these three drugs, suggesting that they may act on novel targets to kill worms. Sertraline, paroxetine, and chlorpromazine have long histories of use clinically as antidepressant or antipsychotic medicines. They may represent new classes of anthelmintic drug that could be used in combination with existing front-line drugs to boost effectiveness of anti-parasite treatment as well as offset the development of parasite drug resistance.

## Introduction

More than 1 billion people world-wide are estimated to be infected with parasitic helminths, resulting in major economic and personal impacts from the years of healthy life lost to morbidity and premature mortality^1,2^. Effects of chronic helminth infection particularly impact pregnant mothers and children, resulting in worsened birth outcomes, anemia, developmental delays, stunting, and educational challenges. Chemotherapeutic control of helminth infections has relied exclusively on the use of a single drug or related classes of drugs over extended periods of time, a strategy that supports selection of anthelmintic-resistant parasites. In fact, resistance to the existing front-line drugs has been detected in livestock and companion animals, and is likely to occur in human helminths as well^3,4^. The identification of new pharmaceutical agents that can cheaply and effectively treat parasitic helminth infections is a critical global health need. However, the journey from initial development to approval for use in humans is long and costly, and it is estimated to exceed 2.5 billion dollars to bring a single new drug to market^5^. Many consumers of anthelmintic drugs are in poor countries with inadequate infrastructure and public health budgets, requiring a low price point per dose to put medication within reach. This means there is likely to be a lower return on investment for the cost of developing a new drug despite the large number of people that would utilize and benefit from it. Repurposing existing clinically approved drugs for anthelmintic use could significantly decrease the cost to develop and translate a new drug to the clinic.

High-throughput compound screening in parasitic helminths is impractical due to the associated costs as well as the difficulties of culturing and maintaining populations of parasites with multiple life-stages requiring different growth environments, including time within a mammalian host. Therefore, developing low-cost, high-throughput screening platforms to identify lead compounds is critical. Identifying compounds that kill *Caenorhabditis elegans*, a non-parasitic soil dwelling nematode, can help direct researchers toward drugs that also kill parasitic helminths. Front-line anthelmintic drugs can kill *C. elegans*, and *C. elegans* have been used to identify novel lead compounds for additional study as well as to dissect the target pathways of known anthelmintic drugs^6,7^. *C*. *elegans* are amenable to low- and high-throughput screens, have a rapid life cycle, and are easy to maintain in a laboratory setting. Therefore, *C. elegans* provides a cost-effective and rapid screening tool to identify new anthelmintic candidates, which can then be followed by testing on targeted parasitic species.

Adopting this strategy, we used *C. elegans* for first-pass screening of the NIH Clinical Collection, a library of small molecules with histories of use in humans. These compounds have associated data pertaining to safety, bioavailability, and dosage, which will expedite repurposing for new indications. From these screens, we identified three drugs that effectively kill *C. elegans* at multiple life stages. Furthermore, these drugs are toxic to three parasitic helminth species: the whipworm, *Trichuris muris*; the hookworm, *Ancylostoma caninum;* and the blood fluke flatworm, *Schistosoma mansoni*. Analysis of *C. elegans* strains with mutations in specific biochemical pathways suggest that the drugs may act via novel pathways, potentially representing new classes of anthelmintics that can be rapidly translated into the clinic.

## Results

### Sertraline, paroxetine, and chlorpromazine are toxic to multiple *C. elegans* developmental stages

To identify novel anthelmintic drug candidates, we surveyed 281 compounds from the NIH Clinical Collection for activities that inhibited *C. elegans* hatching, development, growth, and/or survival. From this screen, we identified 13 FDA-approved drugs that reproducibly caused rapid death or early developmental arrest of *C. elegans* (Supplementary Table S1). These drugs have diverse clinical indications, including uses as anti-arrhythmia, anti-bacterial, anti-cancer, anti-depressant, anti-fungal, and anti-psychotic agents. We selected sertraline, paroxetine, and chlorpromazine for follow-up testing based on their reported tolerability, oral bioavailability, low toxicity, and limited side effect profiles in humans.

Parasitic helminths are typically present in several developmental stages in an infected human, and efficacy against more than one parasite life stage can reduce the number of treatments required or prevent the need to use a combination drug therapy targeting different life stages. Accordingly, we tested the drugs at a range of concentrations in multiple *C. elegans* life stages: embryos, larvae, and fully developed, fertile adults. Worms were placed on solid agar plates with the indicated concentrations of drug (Table 1) and scored using a partially quantitative severity scoring system for effects on motility, development, and survival. Worm populations were assigned a phenotypic description after 48 h of drug exposure. Each negative phenotype observed was assigned a point and these were summed to obtain a total severity score for each drug and concentration tested, similar to a recently reported health-rating system developed for *C. elegans* anthelmintic screening^8^. We found that sertraline, paroxetine, and chlorpromazine all had concentration-dependent, deleterious effects on development, motility and survival at all life stages tested (Table 1).

**Table 1:** Drug effect severity scores for *C. elegans*. *C. elegans* were grown in the presence of the indicated concentrations of drug and scored for visible phenotypes.

**Embryos**	**DMSO**	**5 μM**	**25 μM**	**50 μM**	**75 μM**	**100 μM**	**125 μM**	**150 μM**
Sertraline	0	0	1	4	4	4	4	4
Paroxetine	0	0	1	3	4	4	4	4
Chlorpromazine	0	0	0	1	3	4	4	4
**L1**	**DMSO**	**5 μM**	**25 μM**	**50 μM**	**75 μM**	**100 μM**	**125 μM**	**150 μM**
Sertraline	0	0	1	4	4	4	4	4
Paroxetine	0	0	1	1	4	4	4	4
Chlorpromazine	0	0	0	0	2	2	4	4
**L3/L4**	**DMSO**	**5 μM**	**25 μM**	**50 μM**	**75 μM**	**100 μM**	**125 μM**	**150 μM**
Sertraline	0	0	0	2	2	3	4	4
Paroxetine	0	0	0	0	1	2	4	4
Chlorpromazine	0	0	0	0	0	1	1	4
**Adults**	**DMSO**	**5 μM**	**25 μM**	**50 μM**	**75 μM**	**100 μM**	**125 μM**	**150 μM**
Sertraline	0	0	1	3	4	4	4	4
Paroxetine	0	0	0	1	4	4	4	4
Chlorpromazine	0	0	0	0	4	4	4	4

All treatments were performed in duplicate wells, *N* = 50–100 worms/well, with each experiment repeated at least 3 times. Points were assigned for each scored effect based on the scoring descriptors below after 48 h of drug exposure and additive for each phenotype observed, with a maximum allowed total score of 4. Scoring descriptors were assigned when the entire test population exhibited the phenotype. Arrested = 1; Developmental delay = 1; Lethargic = 1; Paralyzed = 1; Toxic (>90% lethality) = 2; Dead = 4.

**Figure 1:**
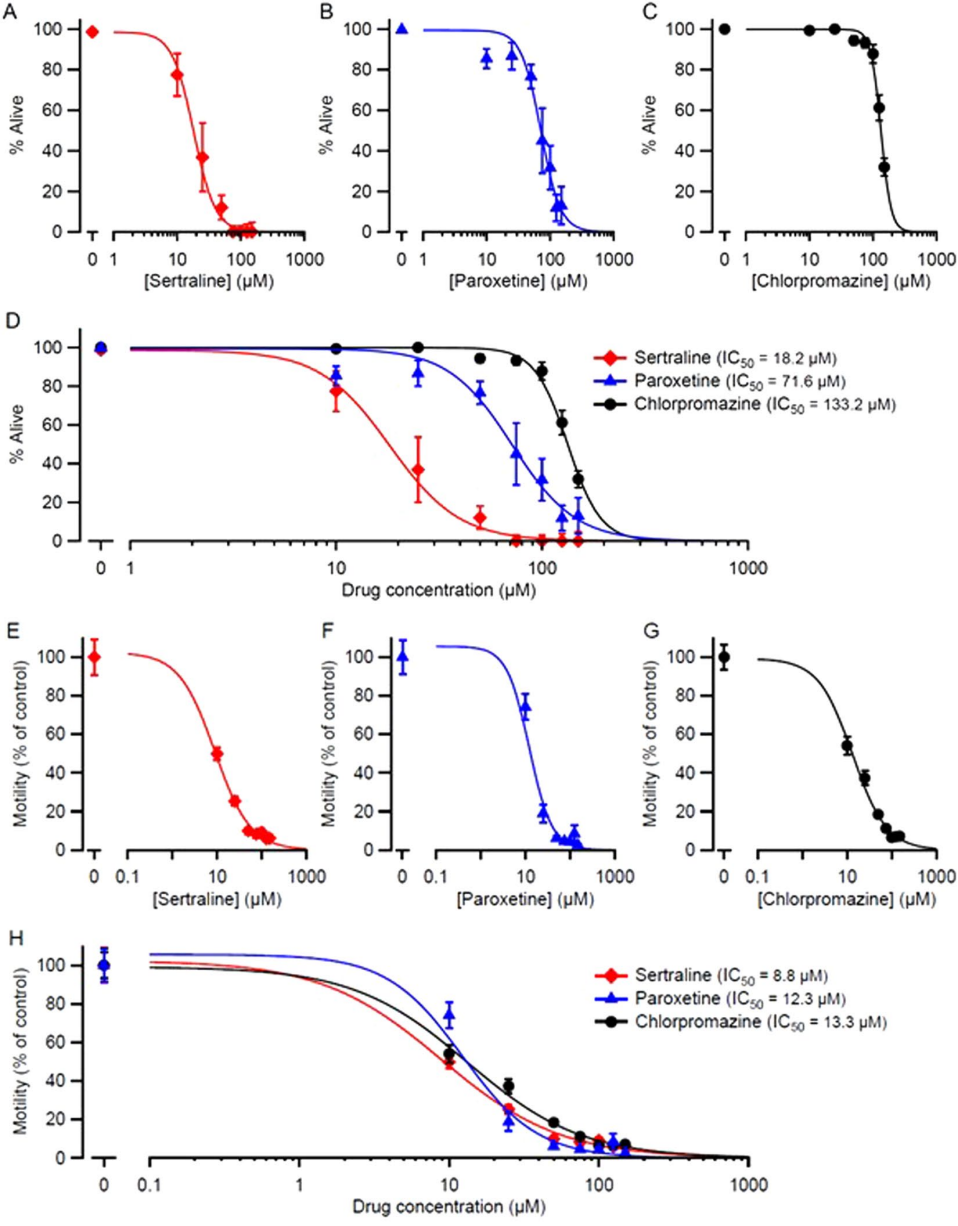
Sertraline, paroxetine, and chlorpromazine impair survival and motility in *C. elegans*. (**A**,**B**,**C**) Embryos were placed on plates containing the indicated concentrations of drug, and hatched embryos were scored at 48 h for survival (mean ± S.E.M.; *N* = 6 replicates; 50–100 embryos plated per replicate). In every case, there was a significant effect of the drug on survival (*p* < 10^−10^; Likelihood Ratio test). (**E**,**F**,**G**) Day 1 adult worms were exposed to sertraline, paroxetine, or chlorpromazine on plates for 24 h. Worm movements were video recorded and movement speeds calculated using automated tracking analysis software (WormLab). Data are shown as mean ± S.E.M.; *N* = 3 replicates; > 40 individual worms tracked per concentration. In every case, there was a significant effect of the drug on motility (*p* < 10^−10^; Likelihood Ratio test). Smooth curves in (**A**–**H)** are best fits to the Hill equation (see Materials and Methods). (**D)** and (**H**) show the superimposition of the curves in (**A**–**C)** and (**E**–**G**), respectively, for comparison. Calculated IC_50_ values (the drug concentration at which survival or motility were reduced by 50%) are shown. Pairwise comparisons of IC_50_ values for lethality showed significant differences between all 3 three drugs (*p* < 10^−8^; Likelihood Ratio test; **D**), but there were no significant differences in IC_50_ for the motility assay between drugs (*p* > 0.08; **H**).

Additional quantitative data were obtained by scoring the percentage of live worms 48 h after exposing embryos to drugs. All three drugs had concentration-dependent effects on survival (Fig. 1A–D). Comparison of IC_50_ values (the concentration of drug that caused 50% lethality) showed that the order of potency was sertraline > paroxetine > chlorpromazine. We also observed strong developmental delay or arrest at 48 h after exposure to the drugs (Supplementary Table S2). To quantify drug effects on *C. elegans* motility, we utilized automated video worm tracking software. Day 1 adult worms were placed on plates containing the indicated concentrations of drug for 24 h, at which point unstimulated locomotion was recorded and analyzed. All three drugs caused concentration-dependent decreases in motility, with similar potency (Fig. 1E–H).

### Sertraline, paroxetine, and chlorpromazine act through non-canonical targets to kill *C. elegans*

Sertraline and paroxetine are selective serotonin (5-hydroxytryptamine; 5-HT) reuptake inhibitors (SSRIs), used clinically as anti-depressant medications. These drugs are known to inhibit the 5-HT reuptake transporter SCL6A4, resulting in an increase in the amount of extracellular 5-HT and increased 5-HT receptor activation^9^. Sertraline has been shown to reduce schistosomulum viability in the trematode parasite, *S. mansoni*^10^. In *C. elegans*, *S. mansoni*, *Ascaris suum* (pig roundworm), and some plant nematodes, treatment with 5-HT can cause altered movement or paralysis^11–14^, whereas in many free living and parasitic nematodes 5-HT stimulates feeding behavior^15–17^.

Drugs that perturb serotonergic signaling thus have potential as anthelmintics, but it is important to identify the molecular targets upon which the drugs act. To test the role of specific genes in the anthelmintic activity of these drugs, mutant and wild-type *C. elegans* embryos were placed on solid agar plates with a range of drug concentrations effective on wild-type *C. elegans*, and scored semi-quantitatively for positive or negative effects on population development, motility and survival compared to wild-type worms. The toxicity of sertraline and paroxetine could potentially result from their canonical SSRI activities, acting on the *C. elegans* functional homolog of SCL6A4, MOD-5^18^, and increasing bioavailable 5-HT. To test this hypothesis, we determined whether a *mod-5* loss of function mutant was resistant to the effects of sertraline and paroxetine. Interestingly, we found that *mod-5(n822)* mutant worms were more sensitive to sertraline and paroxetine than wild-type controls (Supplementary Table S3), suggesting that these drugs acted via another target or targets in *C. elegans*. We also tested whether the *C. elegans* gene with the next highest sequence homology to SCL6A4, the dopamine (DA) transporter *dat-1*, was important for mediating drug susceptibility. We found that the *dat-1* loss-of-function mutation *dat-1(ok157)* was not more resistant to sertraline or paroxetine than wild-type controls (Supplementary Table S3). In addition, we tested loss of function mutations in *tph-1(n4622)*, tryptophan hydroxylase required for 5-HT biosynthesis; four identified serotonin-binding receptors in *C. elegans*: *mod-1(ok103)*, *ser-4(ok512)*, *ser-7(tm1325)* and *ser-1(ok345)*; and *glr-1(n2641*), an AMPA receptor that functions downstream of *ser-7*. None of these mutants exhibited differences in susceptibility to sertraline or paroxetine compared to wild-type worms, despite their key roles in 5-HT synthesis or signaling (Supplementary Table S3).

A number of *C. elegans* mutants have been generated previously with resistance to a nose-retraction phenotype in the presence of low doses of fluoxetine (Prozac), another SSRI antidepressant^19^. Two of these mutants were mapped to specific genetic loci and characterized for their roles in *C. elegans*^19,20^. One of them, *nrf-5*, encodes a lipid-binding protein involved in hydrophobic molecule transport in the intestine, while the other, *nrf-6*, is involved in embryonic viability and yolk transport. We tested whether these fluoxetine-resistant mutants were also resistant to sertraline and paroxetine. The survival of *nrf-5(sa513)* worms in these drugs was unchanged from wild-type *C. elegans*. Interestingly, *nrf-6(sa525)* worms were more sensitive than wild-type to paroxetine, although not to sertraline (Supplementary Table S3). The finding that mutations in the genes listed in Supplementary Table S3 failed to reduce the anthelmintic activity of sertraline and paroxetine in *C. elegans* worms suggested that one or more other targets of these drugs underlie their anthelmintic effects.

Chlorpromazine gained widespread use in the 1950’s as the first neuroleptic drug, and is thought to primarily act through antagonism of DA D2 receptors^21^. Chlorpromazine has been reported to decrease embryonic survival in *C. elegans* and *Haemonchus contortus* (a parasitic nematode of ruminants)^22^, kill L3 larvae of *Ancylostoma ceylanicum* (hookworm)^23^, and reduce the motility of *Dirofilaria immitis* (canine heartworm)^24^. Exogenously applied DA can also reduce movements of *Meloidogyne incognita* and *Heterodera glycines*, two parasitic nematodes of plants^11^. As with SSRI drugs, the molecular target or targets that underlie the anthelmintic activity of chlorpromazine have not been identified.

To test the hypothesis that D2 receptor antagonism is required for the anthelmintic activity of chlorpromazine, we tested its effects on *C. elegans* with loss of function mutations in both D2-like receptors, DOP-2 and DOP-3. We found that the *dop-2(vs105); dop-3(vs106)* mutant worms were not more resistant to chlorpromazine than wild-type *C. elegans* (Supplementary Table S3). We also tested loss of function mutations in *cat-2(e1112)*, tyrosine hydroxylase required for DA synthesis, and *dat-1(ok157)*, the *C. elegans* DA transporter. *cat-2(e1112)* worms were more sensitive to chlorpromazine, while *dat-1(ok157)* and wild-type worms had similar sensitivity to chlorpromazine. These findings suggested that intact DA signaling was not required for this drug’s anthelmintic activity.

Chlorpromazine has a number of other known targets, including 5-HT, histamine, α-adrenergic, and muscarinic receptors^25^. To begin exploring these candidate pathways, we tested the sensitivities of the 5-HT signaling mutants *tph-1(n4622)*, *mod-1(ok103)*, *mod-5(n822)*, *ser-4(ok512)*, and *ser-7(tm1325)*; *ser-1(ok345)* to chlorpromazine, but none exhibited any increased resistance to chlorpromazine (Supplementary Table S3). In summary, the finding that mutations in the genes listed in Supplementary Table S3 failed to reduce the ability of chlorpromazine to kill *C. elegans* worms suggests that one or more other targets of this drug underlies its anthelmintic effects.

Potentially, the anthelmintic activity of sertraline, paroxetine and chlorpromazine on *C. elegans* might be mediated through other neurotransmitter signaling pathways. Therefore, we tested whether genetic disruption of octopamine (OA) or tyramine (TYR) signaling would alter worms’ susceptibilities to these drugs. We tested the TYR receptor *ser-2(pk1357)*; two alleles of the OA receptor *ser-3 (ok2007* and *ad1774)*; the TYR decarboxylase required for synthesis of TYR and OA, *tdc-1(n3419)*; and the TYR beta-hydroxylase required for TYR synthesis, *tbh-1(n3247)*. However, none of these mutants were less sensitive than controls to sertraline, paroxetine, or chlorpromazine (Supplementary Table S3).

*C. elegans* have been used to dissect anthelmintic drug mechanisms of action, primarily through the selection of drug-resistant mutants, leading to identification of key target pathways. We expected that, if sertraline, paroxetine or chlorpromazine acted on the same targets as known anthelmintic drugs, mutants resistant to the known anthelmintics should likewise exhibit resistance to sertraline, paroxetine and/or chlorpromazine. Therefore, we tested mutants resistant to emodepside (*slo-1(js379)*), *Bacillus thuringiensis* (BT) toxin (*bre-1(ve4)*), levamisole (*unc-29(e193)* and *unc-50(e306)*), monepantel (*acr-23(ok2804)*), ivermectin (*avr-14(ad1305);avr-15(vu227)*;*glc-1(pk54)*), or benzimidazole (*ben-1(e1880)*)^26–31^ representing a broad range of anthelmintic targets and modes of action. However, none of the resistance mutants tested were more resistant to sertraline, paroxetine or chlorpromazine than wild-type worms (Supplementary Table S3), suggesting that the anthelmintic activities of these drugs on *C. elegans* may be mediated via previously unidentified targets or pathways.

### Sertraline, paroxetine, and chlorpromazine rapidly inhibit *C. elegans* pharyngeal pumping

Many existing anthelmintics act via ion channels or neurotransmitter receptors^32^. Sertraline, paroxetine and chlorpromazine are all candidates for this mode of action, even if their specific targets are as yet unknown. To test whether these drugs exerted rapid effects on electrical signaling, we used a microfluidic device (‘chip’) to record electropharyngeograms (EPGs) from *C. elegans* adults while perfusing control or drug solutions over the worms^16,33^. This non-invasive method records the electrical currents emitted by pharyngeal muscles and neurons during pharyngeal pumping (feeding behavior). 5-HT was included in the perfused solutions to induce robust, sustained pharyngeal pumping, providing a baseline against which feeding-inhibitory effects could be quantified. Known anthelmintic drugs that act on ion channels or neurotransmitter receptors rapidly inhibit EPG activity^16,33^.

**Figure 2:**
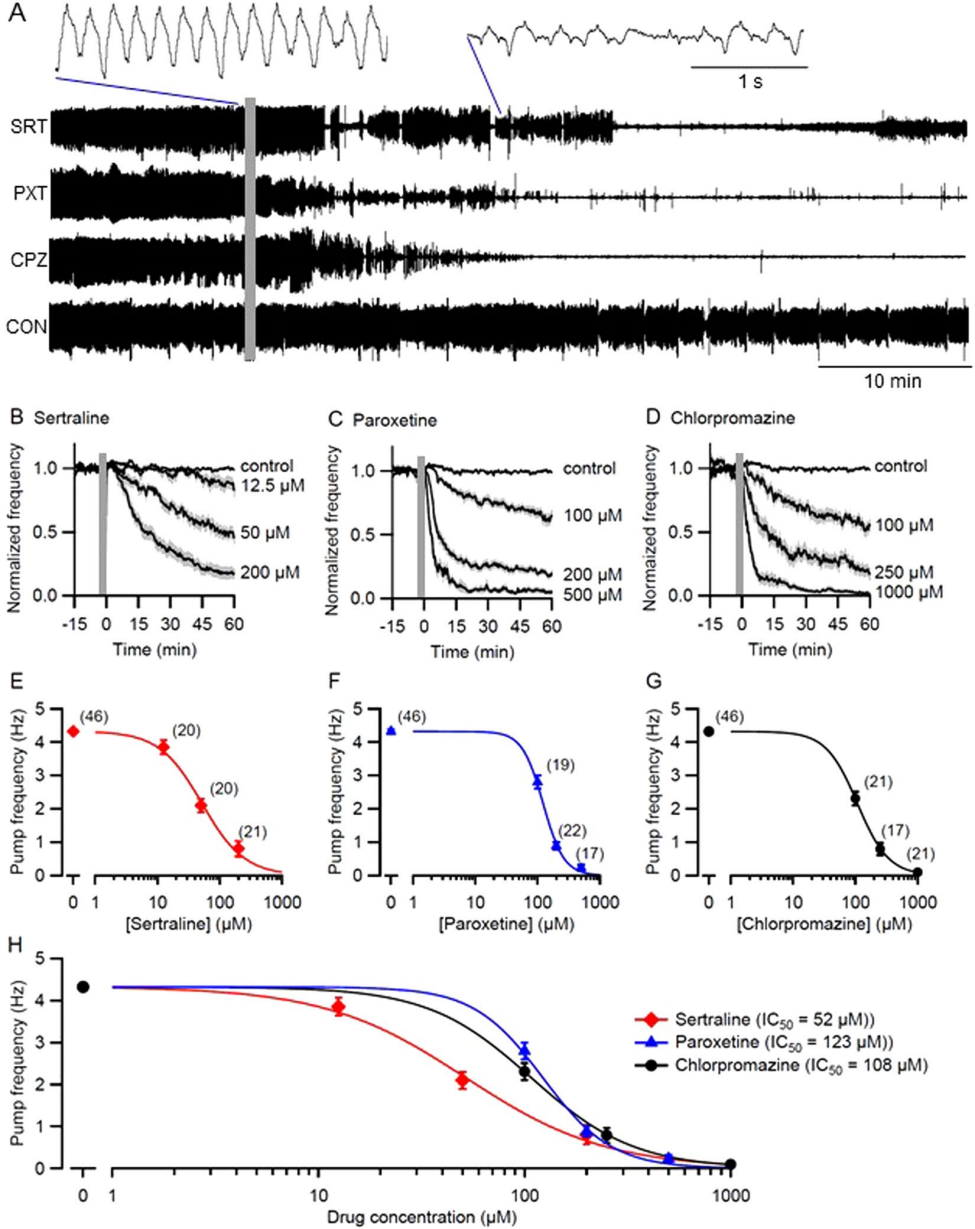
Sertraline, paroxetine and chlorpromazine inhibit pharyngeal pumping in *C. elegans*. Pumping in day 1 adults was monitored by EPG recordings obtained in a microfluidic chip, in M9 buffer containing 10 mM 5HT (M9-5HT). After 30 min perfusion with M9-5HT the perfusate was switched (grey bars) to M9-5HT containing drug or solvent. Only a portion of the baseline recording period is shown. (**A**) Excerpts of representative EPG recordings from 4 individual worms switched to (top to bottom): 200 µM sertraline (SRT); 500 µM paroxetine (PXT); 1000 µM chlorpromazine (CPZ); and 0.5% DMSO (CON). In these time-compressed recordings, individual pumps are not visible and pumping appears as a thick, dark line. Insets at an expanded time base (top) show the characteristic decrease in pump frequency and EPG amplitude caused by all 3 drugs. (**B**,**C**,**D**) Concentration- and time-dependence of pump inhibition by SRT, PXT and CPZ, compared to controls. Pump frequency was normalized to the frequency prior to switching perfusate and displayed as mean (line) ± S.E.M. (shading). (**E**,**F**,**G**) Pump frequency from (**B**,**C**,**D)** was plotted against drug concentration at *t* = 55–60 min (mean ± S.E.M.) and fit using the Hill equation. Number of worms per point (*N*) shown in parentheses. In every case, there was a significant effect of the drug on pump frequency (*p* < 10^−10^; Likelihood Ratio test). (**H**) Superimposition of the curves in (**E**,**F**,**G**) for comparison. Calculated IC_50_ values (the drug concentration at which pump frequency was reduced by 50%) are shown. The IC_50_ value for sertraline differed significantly from the values for paroxetine (*p* < 10^−7^) and chlorpromazine (*p* < 10^−4^) whereas chlorpromazine and paroxetine IC50s did not differ significantly (*p* > 0.2; Likelihood Ratio test).

Continuous EPG recordings were taken while switching from the control solution to a drug or control solution (Fig. 2). Drug concentrations were selected to span a range of effectiveness suitable for determining IC_50_ values (the concentration at which pumping frequency was reduced by half). We found that all three drugs reduced the frequency of pharyngeal pumping, or terminated it completely, while control worms continued to pump at the control rate (Fig. 2A). The drugs also diminished the amplitude of EPG signals compared to controls (insets, Fig. 2A). Inhibition of pharyngeal pumping by sertraline, paroxetine and chlorpromazine was rapid—sometimes within 5 min—and concentration-dependent (Fig. 2B,C,D). The rapidity of the inhibition was consistent with perturbation of ion channels or neurotransmitter receptors. Dose-response curves (Fig. 2E,F,G,H) and statistical comparison of IC_50_ values showed that sertraline was the most potent, with paroxetine and chlorpromazine having similar potencies. Taken together, these results suggest that disruption of electrical signaling, and potentially the disruption of feeding behavior, could contribute to the lethality of sertraline, paroxetine and chlorpromazine. In these experiments, all three drugs inhibited pharyngeal pumping in the presence of a relatively high level of 5-HT (10 mM); this finding is consistent with the results of the mutant analysis above (Supplementary Table S3) which likewise suggested that the drugs’ deleterious effects were not mediated by an increase in 5-HT levels.

### Sertraline, paroxetine, and chlorpromazine decrease motility of the parasitic whipworm *Trichuris muris*

**Figure 3:**
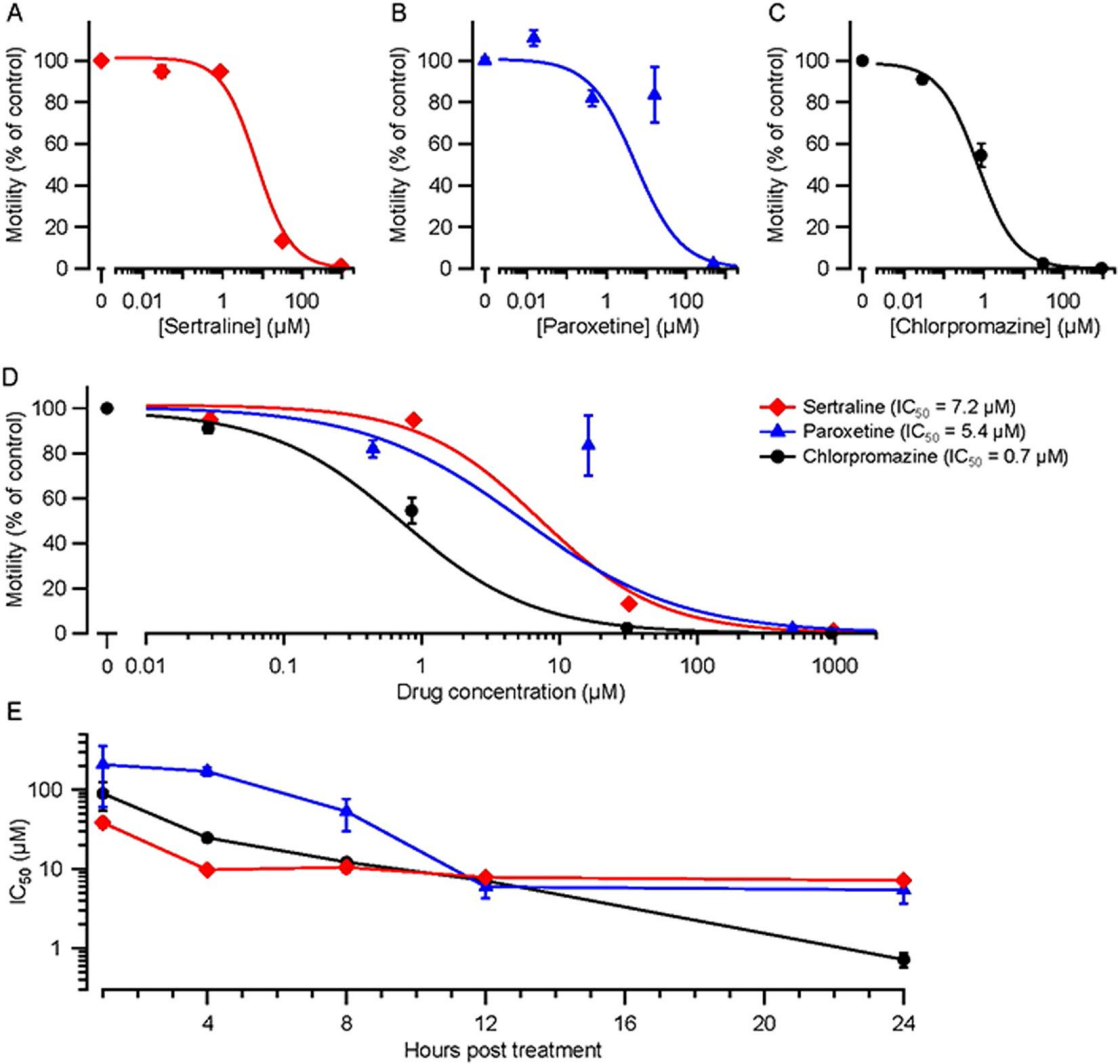
Sertraline, paroxetine and chlorpromazine inhibit motility of adult *T. muris*. (**A**,**B**,**C**) Adult worms were cultured in different concentrations of sertraline, paroxetine or chlorpromazine for 24 h, after which their movements were videotaped and analyzed. Motility was normalized to the motility of medium-only, and medium with 1% DMSO, controls at 24 h. Data are shown as mean ± S.E.M.; *N* = 3 replicates; 3 worms per replicate. In every case, there was a significant effect of the drug on motility (*p* < 10^−10^; Likelihood Ratio test). (**D**) Superimposition of the curves in (**A**,**B**,**C**) for comparison. Calculated IC_50_ values (the drug concentration at which motility was reduced by 50%) at 24 h are shown. At 24 h, the IC_50_ value for chlorpromazine differed significantly from the values for sertraline (*p* < 10^−10^) and paroxetine (*p* < 10^−5^) whereas sertraline and paroxetine IC_50_s barely differed (*p* = 0.48; Likelihood Ratio test). (**E**) IC_50_ values for *T. muris* motility computed at four earlier time points (1, 4, and 12 h) in addition to the 24-h data in (**A**–**D**). The same worms were analyzed at each time point and IC_50_s were calculated from Hill plots (not shown). At 1 h post-treatment, the IC_50_ of sertraline differed significantly from the values for paroxetine (*p* = 0.006) and chlorpromazine (*p* = 0.04), whereas the IC_50_s of paroxetine and chlorpromazine did not differ significantly (*p* = 0.26; Likelihood Ratio test).

Although many studies have utilized *C*. *elegans* as a surrogate screening platform for anthelmintic compounds, it is not clear which parasitic species are best modeled by *C*. *elegans* nor whether a novel intervention that kills *C*. *elegans* will be effective in other species. Therefore, we tested the effects of sertraline, paroxetine and chlorpromazine against *Trichuris muris*, a whipworm parasite of mice similar to the human parasite *Trichuris trichiura. T. muris* adults were isolated from infected mice, maintained *in vitro* in culture dishes, exposed to drugs, and monitored every 4 h for 24 h. Parasite movements were captured by video and analyzed for changes in motility over time. All three drugs produced concentration-dependent reductions in motility, as shown in Fig. 3A,B,C,D for the 24 h time point. Statistical comparison of IC_50_ values showed that, at 24 h, chlorpromazine was the most potent in reducing motility compared to sertraline and paroxetine. Figure 3E presents IC_50_ data for additional times post-treatment. As expected, IC_50_ values decreased as exposure time increased. For the first 8 h of exposure, the order of potency was sertraline > chlorpromazine > paroxetine. At 12 h post-treatment, the IC_50_s were similar. Finally, at 24 h, chlorpromazine was significantly more effective than either other drug in impairing motility.

### Sertraline, paroxetine, and chlorpromazine are toxic to the hookworm *Ancylostoma caninum*

**Figure 4:**
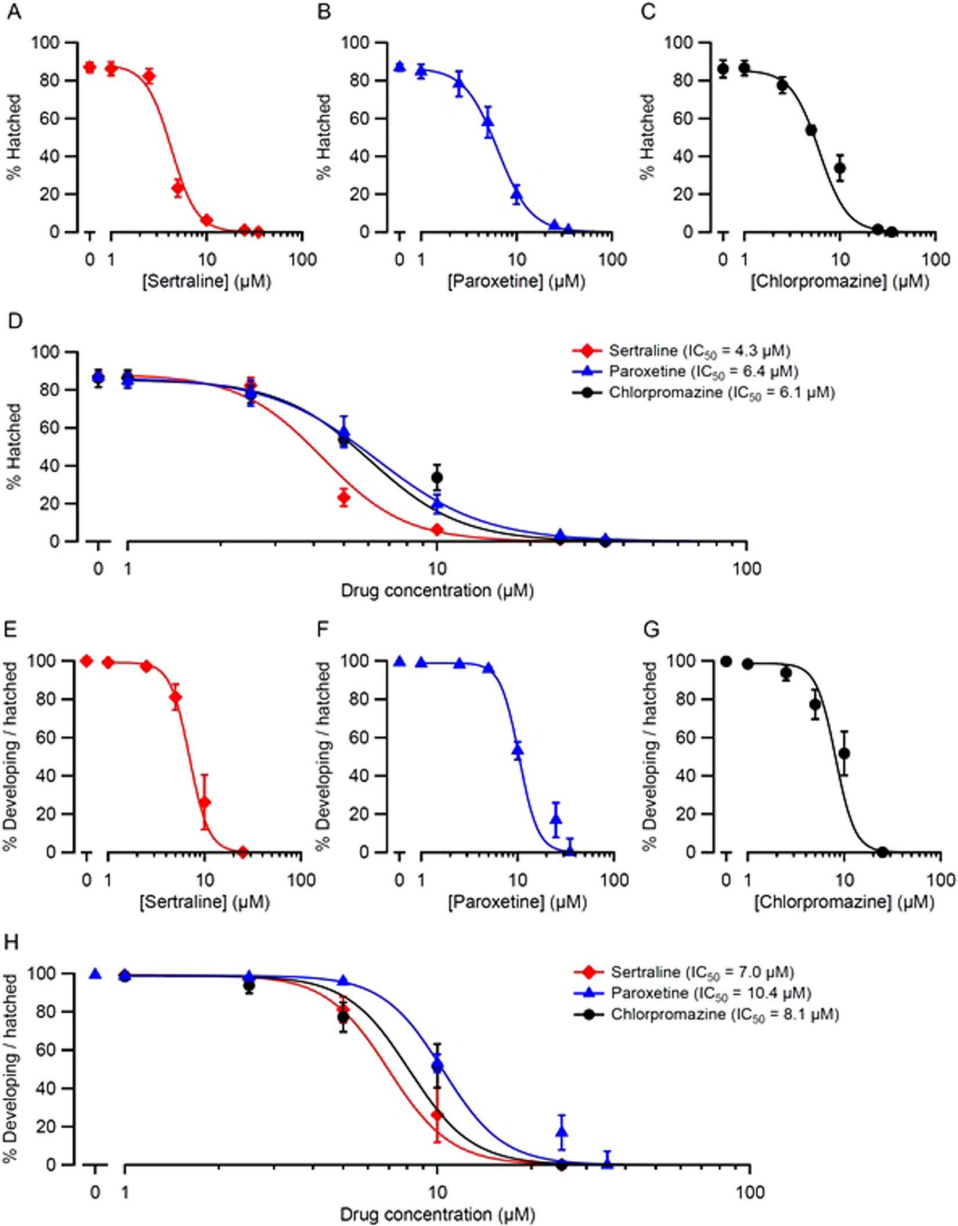
Sertraline, paroxetine and chlorpromazine impair hatching and development of *A. caninum*. Hookworm eggs were placed on plates containing the indicated concentrations of drug and scored for hatching and development after 4 d. The results of two pooled experiments (*N* = 6 replicates, 100 eggs per replicate) for each drug at each concentration are represented two ways. (**A**,**B**,**C**) Percentage of eggs that hatched. (**E**,**F**,**G**) Percentage of hatched worms that developed beyond the L1 stage. Panels D and H show the plots in (**A**–**C** and **E**–**G**), respectively, superimposed for comparison. Calculated IC_50_ values (the drug concentration at which hatching or development were reduced by 50%) are shown. The IC_50_ for percent hatched in sertraline differed significantly from the IC_50_s for chlorpromazine (*p* < 10^−6^) and paroxetine (*p* < 0.002; **D**). The IC_50_ for percent developing per hatched in sertraline differed significantly from paroxetine (*p* < 0.002; **H**). All other IC_50_ comparisons were not significantly different (*p* > 0.4; Likelihood Ratio test).

We next tested the effects of sertraline, paroxetine and chlorpromazine on the hookworm, *Ancylostoma caninum. A. caninum*, a significant pathogen of dogs and a commonly-used laboratory surrogate for human hookworms, is highly diverged from *T. muris*. *A. caninum* embryos were isolated from infected dogs and cultured in the presence of a range of concentrations of sertraline, paroxetine or chlorpromazine. After 24 h the worms were scored for hatching and development in the presence of drug (Fig. 4). We found that *A. caninum* embryos exposed to sertraline, paroxetine or chlorpromazine exhibited concentration-dependent failure to hatch (Fig. 4A,B,C), with sertraline being more potent than paroxetine or chlorpromazine (Fig. 4D). In embryos that were able to hatch, all three drugs caused concentration-dependent inhibition of development beyond the L1 stage (Fig. 4E,F,G). The three drugs had generally similar potencies, with sertraline being the most potent in this assay (Fig. 4H) as well as in the hatching assay (Fig. 4D). In summary, all three drugs significantly inhibited the hatching and development of *A. caninum* eggs.

### Sertraline, paroxetine, and chlorpromazine kill the parasitic flatworm *Schistosoma mansoni*

**Figure 5:**
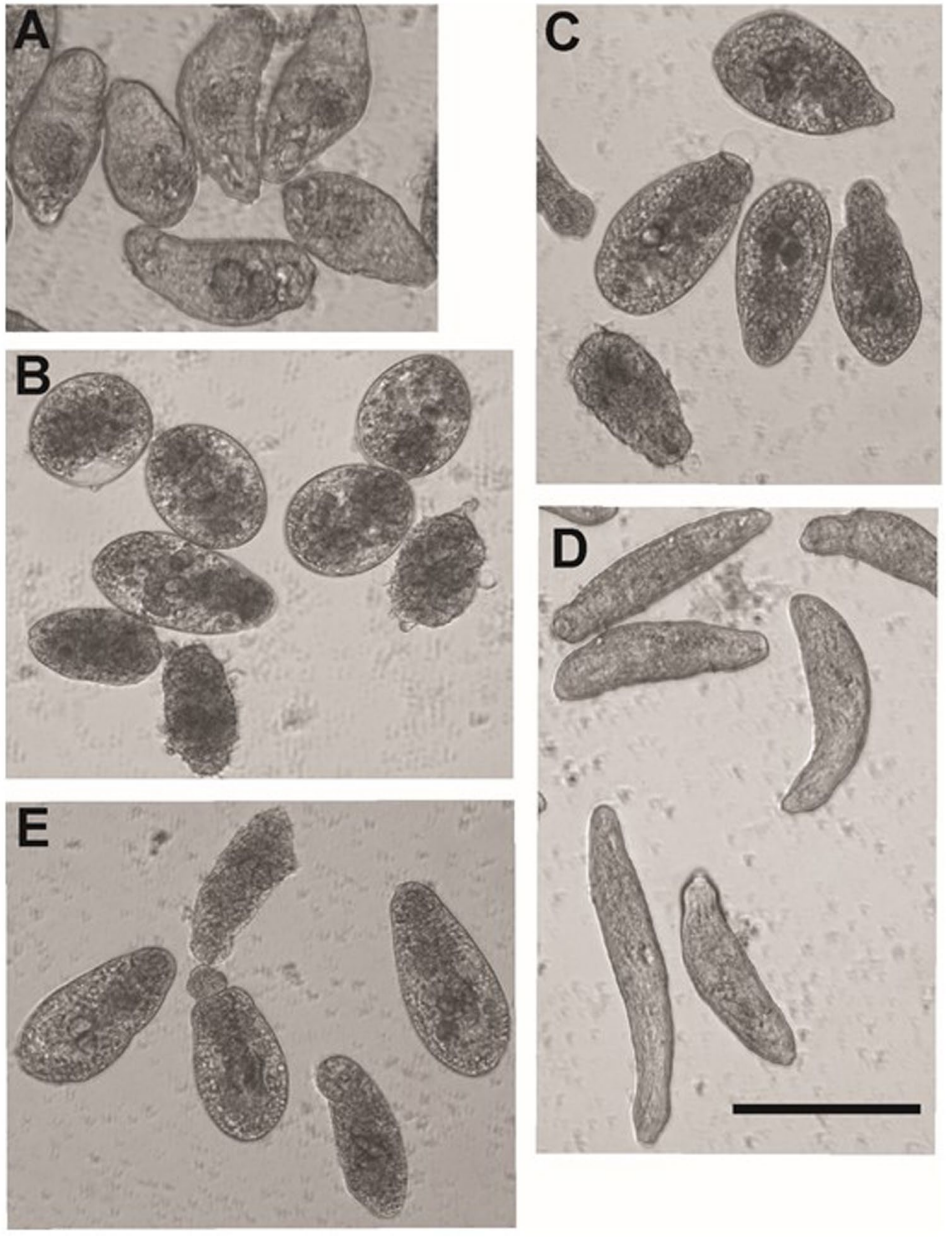
Phenotypic changes in *S. mansoni* somules (post-infective larvae) following incubation with sertraline, paroxetine or chlorpromazine. Images were obtained after 48 h in culture with either DMSO (control) or the indicated concentrations of drugs. (**A**) Control somules. (**B**) 5 µM sertraline produced rounding, degeneration and death. (**C**) 5 µM paroxetine produced rounding and degeneration. (**D**) 5 µM chlorpromazine produced somule lengthening and overactivity, whereas (**E**) 10 µM chlorpromazine produced rounding and degeneration. Scale bar = 200 µm. Additional phenotypic data are given in Table 2.

To examine the effects of sertraline, paroxetine and chlorpromazine on a third parasitic helminth species, we tested them on the blood fluke, *Schistosoma mansoni*. This schistosome species is prevalent in Africa and South America, and is highly divergent from the nematodes, *C. elegans*, *T. muris* and *A. caninum*. *S. mansoni* somules (post-infective larvae in the mammalian host) were isolated, exposed to sertraline, paroxetine or chlorpromazine and surveyed at 18 and 48 h post-treatment for morphological changes and survival. Using a partially quantitative severity scoring system based on the phenotypes observed, including changes in opacity and shape, impaired motility, impaired sucker adherence, degeneration and death, all three drugs caused dramatic, time- and concentration-dependent morphological changes in somules (Table 2; Fig. 5). Sertraline and paroxetine produced the most severe effects.

**Table 2:** Drug effect severity scores for *S. mansoni*. *S. mansoni* somules or adult males were incubated for the indicated durations and concentrations of drug, and scored for phenotypic changes.

Somules	18 hours						48 hours					
	DMSO	0.625 μM	1.25 μM	2.5 μM	5 μM	10 μM	DMSO	0.625 μM	1.25 μM	2.5 μM	5 μM	10 μM
Sertraline	0	0	0	1	4	4	0	0	0	1	4	4
Paroxetine	0	2	2	2	4	4	0	2	2	4	4	4
Chlorpromazine	0	2	2	2	2	4	0	2	2	2	2	4
**Adult Males**	**11 hours**						**24 hours**					
	**DMSO**	**0.625 μM**	**1.25 μM**	**2.5 μM**	**5 μM**	**10 μM**	**DMSO**	**0.625 μM**	**1.25 μM**	**2.5 μM**	**5 μM**	**10 μM**
Sertraline	0	0	0	0	0	4	0	0	0	0	2	4
Paroxetine	0	1	1	1	1	4	0	1	1	1	1	4
Chlorpromazine	0	0	1	1	1	2	0	0	1	1	1	3

Assays were performed in duplicate wells with either 40 somules or 5 adult male worms per well. Data from one of two experiments are shown. Each phenotypic descriptor observed was awarded a score of 1 and the scores were added up to a maximum score of 4, as indicated below. The most severe descriptors received a score of 4. Descriptors and their scores were assigned when the entire test population exhibited the phenotype. Dark = 1; Male suction impaired = 1; Overactive = 1; Rounded = 1; Slow = 1; Uncoordinated = 1; Degenerating = 4; Tegument damage (adults) = 4; Dead = 4.

**Figure 6:**
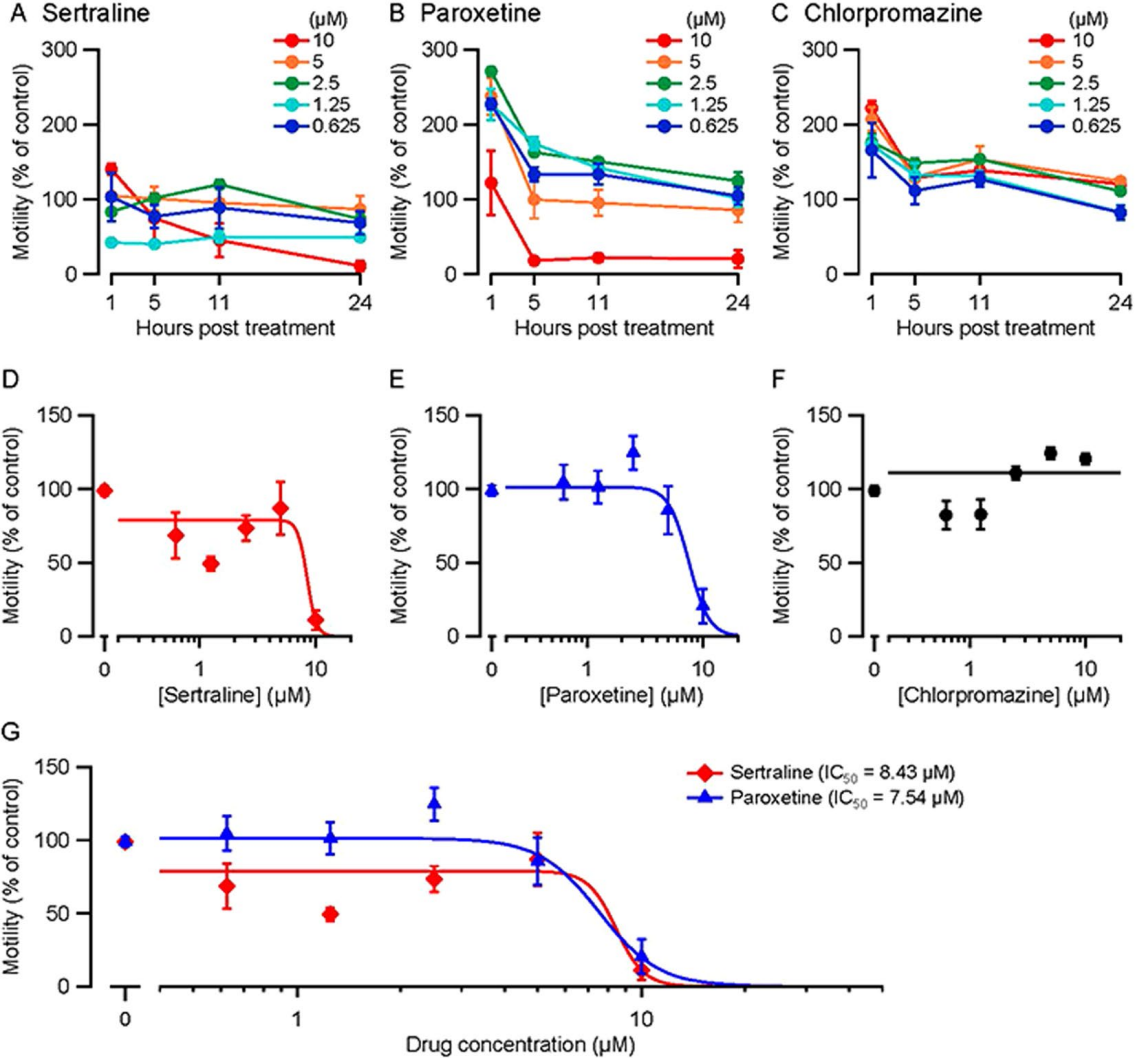
Effects of sertraline, paroxetine and chlorpromazine on motility of adult *S. mansoni*. Adult male worms were cultured in the presence of sertraline, paroxetine, chlorpromazine or DMSO at the indicated concentrations. Motility was measured using WormAssay after 1, 5, 11 and 24 h (*N* = 4 replicates, 60 s recording period) and normalized to DMSO controls (set to 100%). (**A**,**B**,**C**) Motility (mean ± S.E.M.) plotted against time for the drug concentrations shown. (**D**,**E**,**F**) Motility plotted against concentration at 24 h (same data as **A**,**B**,**C**). The sertraline and paroxetine data could be fitted by the Hill equation whereas the chlorpromazine data could not (a linear fit is shown). Both sertraline and paroxetine had significant effects on motility at 24 h (*p* < 10^−10^; Likelihood Ratio test). (**G**) Shows the plots from (**D)** and (**E)** superimposed for comparison. Calculated IC_50_ values (the drug concentration at which motility was reduced by 50%) are shown. IC_50_ values for sertraline and paroxetine were not significantly different (*p* = 0.66; Likelihood Ratio test).

We also tested the three drugs on adult *S. mansoni;* degenerative changes were scored at 11 and 24 h (Table 2) and worm motility was measured at 1, 5, 11 and 24 h over five concentrations of drug (Fig. 6). Adults exhibited a range of phenotypes including uncoordinated movements and impaired adherence to the bottom of the culture well via the ventral sucker, with sertraline and paroxetine producing the most severe effects (Table 2). For motility testing of adult *S. mansoni*, the temporal response pattern varied by drug and concentration (Fig. 6A,B,C). Paroxetine and chlorpromazine produced an early phase of hyperactivity that returned toward control levels over time. At 24 h post-treatment, sertraline and paroxetine both caused a concentration-dependent impairment of motility (Fig. 6D,E), whereas chlorpromazine did not (Fig. 6F), even though chlorpromazine caused adverse phenotypic changes (Table 2). In summary, *S. mansoni* somules and adults were most sensitive to sertraline and paroxetine, with chlorpromazine causing less severe effects.

Taken together, the experiments above suggest that anthelmintic activity of sertraline, paroxetine, and chlorpromazine may be conserved across more than 1 billion years of evolution, following the separation of nematodes such as *C. elegans*, *T. muris* and *A*. *caninum* from trematodes like *S*. *mansoni*^34^.

## Discussion

In this study, we found that thirteen FDA-approved drugs in the NIH Clinical Collection library exhibit anthelmintic activity in *C. elegans*. Based on their favorable pharmacological and safety profiles in humans, we selected sertraline, paroxetine (two SSRI inhibitor anti-depressant drugs) and chlorpromazine (a DA D2 receptor antagonist anti-psychotic drug) for further characterization in *C. elegans* and three parasitic helminths. In *C. elegans*, the drugs impaired development and motility, killed multiple life stages and rapidly disrupted pharyngeal pumping. In *T. muris*, the drugs impaired motility of adult worms. In *A. caninum*, the drugs impaired hatching and development. Finally, in *S. mansoni*, the drugs caused degeneration and death of somules, and impaired motility and death of adult worms. Thus, sertraline, paroxetine and chlorpromazine all exhibit significant anthelmintic activity against widely diverged nematode and trematode species.

Table 3 summarizes the IC_50_ values for the three drugs in each species and assay tested in this study. Comparison of IC_50_s of the three drugs in a given assay and treatment duration is informative: e.g., for lethality of *C. elegans* after 48 h, the order of drug potency was sertraline > paroxetine > chlorpromazine (Fig. 1D, Table 3). Comparing IC_50_ values across species and assays is complicated by differences in the phenotypes measured and treatment durations. These limitations aside, several conclusions are possible. For all three assays in *C. elegans*, sertraline had the greatest anthelmintic activity (lowest IC_50_), with paroxetine being the next most effective in two of the three assays (Table 3). Inhibition of pharyngeal pumping in *C. elegans* during a 60 min treatment required much higher drug concentrations, with IC_50_ values ~2–10 times greater than for the lethality and motility assays (Fig. 2H; Table 3). It is likely that lower drug concentrations would have likewise inhibited feeding if applied for longer durations, but the purpose of the EPG recordings was to test whether the drugs can rapidly disrupt electrical signaling, as seen with anthelmintics that target ion channels and neurotransmitter receptors^16,33^. All three drugs inhibited EPG activity within minutes (Fig. 2B,C,D), consistent with this potential mode of action. As discussed below, the relatively impermeable *C. elegans* cuticle may necessitate the use of higher drug concentrations, especially when seeking rapid responses.

**Table 3:** Summary of IC_50_ values for drugs, species and assays.

Species, Assay & Treatment Duration	IC_50_ (μM)		
	Sertraline	Paroxetine	Chlorpromazine
***C. elegans***			
Lethality (48 h)^a^	18.2*	71.6*	133.2*
Motility (24 h)^a^	8.8 ^ns^	12.3 ^ns^	13.3 ^ns^
Pumping inhibition (60 min)^b^	52*	123^ns^	108^ns^
***T. muris***			
Motility (1 h)^c^	38.4*	207.6^ns^	89.5^ns^
Motility (24 h)^c^	7.2^ns^	5.4^ns^	0.7*
***A. caninum***			
Hatching (24 h)^d^	4.3*	6.4^ns^	6.1^ns^
Developing/hatched (24 h)^d^	7.0**	10.4^ns^	8.1^ns^
***S. mansoni***			
Motility (24 h)^e^	8.4 ^ns^	7.5 ^ns^	ND

Source of data: ^a^Fig. 1; ^b^Fig. 2; ^c^Fig. 3; ^d^Fig. 4; ^e^Fig. 6. Underline indicates the drug(s) with the lowest IC_50_ for each test. *Differs significantly from other values in same row; ^ns^values do not differ significantly from other values labeled “ns” in same row; **differs significantly from paroxetine but not chlorpromazine. ND, not determined; an IC_50_ value could not be calculated.

The *T. muris* data (Fig. 3E; Table 3) illustrate the common pharmacological principle that drug effectiveness depends on both concentration and treatment duration: e.g., for motility impairment in *T. muris* adults treated with chlorpromazine, the IC_50_ value for a 1 h treatment was ~ 75 times greater than for a 24 h treatment (Table 3). The temporal pattern of drug effectiveness in *T. muris* was interesting as well. At 24 h, chlorpromazine was clearly the most potent drug in impairing motility, with an IC_50_ approximately 8–10 times smaller than the other two drugs (Fig. 3E, Table 3). In contrast, during the first 12 h of treatment, chlorpromazine’s potency was intermediate between, or similar to, sertraline and paroxetine. The finding that chlorpromazine was most effective as an anthelmintic against *T. muris* when provided at a lower concentration for a longer duration is a favorable property with respect to potential side effects. In *A. caninum*, sertraline had the lowest IC_50_ for both the hatching and development assays, with paroxetine having the highest IC_50_ in both assays (Fig. 4D,H; Table 3). In *S. mansoni*, sertraline and paroxetine had the greatest anthelmintic activity in both somules and adults (Table 2; Fig. 6).

Comparing across species, higher drug concentrations were generally required for anthelmintic effects in *C. elegans* compared to the three parasitic helminths (Table 3). This is expected, as the *C. elegans* cuticle is highly impermeable to environmental small molecules^35^, likely resulting from their evolution as a free-living soil nematode. The concentrations found effective in our study are comparable to other recent anthelmintic screening efforts in *C. elegans*^6,36^

In summary, across four divergent species of worms, sertraline consistently exhibited the highest anthelmintic activity with two exceptions: first, paroxetine was as effective as sertraline in impairing motility of *S*. *mansoni* adults after 24 h and, second, chlorpromazine was most effective in 24 h treatments of *T. muris*. The IC_50_ values of sertraline for 24 h treatments were remarkably similar across the different species examined in this study, varying only 2-fold (4.3, 7.0, 7.2, 8.4 and 8.8 μM; Table 3). Similarity in effective concentration is a favorable property when seeking drug candidates that can kill multiple helminth species at once.

An advantage of potentially repurposing FDA-approved drugs is their existing clinical histories with known dosage, side effect and tolerance profiles. An important consideration is how serum concentrations achieved during traditional clinical use compare with the concentrations tested in this study. The average serum levels for patients taking these drugs clinically are estimated at 58 nM (sertraline), 80 nM (paroxetine), and 422 nM (chlorpromazine)^37,38^, considerably higher than the effective concentrations we determined in this study. For tests with 24 to 48 h treatments, the IC_50_ values ranged from 0.7 to 18.2 µM (Table 3), and are approximately 10 to 300 times the serum levels in psychiatric patients. A caveat in these comparisons is that anthelmintic drug efficacy can differ *in vitro* and *in vivo*^39^. The ability of sertraline, paroxetine and chlorpromazine to reduce worm burdens in mammalian hosts, and at what dosages and administration schedules, remain to be determined. Such experiments are beyond the scope of the present study but are an important next step.

Ideally, clinical administration of anthelmintic drugs is limited to a single or very few doses, which is a treatment protocol that differs considerably from the chronic administration of sertraline, paroxetine, or chlorpromazine for psychiatric purposes. It will be important to test whether treatment with a limited number of higher doses (alone or in combination with other anthelmintic drugs) could eliminate parasitic helminths without adverse side effects. Further, sertraline, paroxetine and chlorpromazine represent psychiatric classes of drugs that have undergone decades of extensive medicinal chemistry efforts (including but not limited to the structural derivative patents referenced here^40–48^). Derivatives of these drugs with poor blood-brain-barrier penetrance may exist that could be effective anthelmintics at lower effective concentrations and without undesirable neurological side effects.

One of the most significant findings of this study concerns the potential mode(s) of action of sertraline, paroxetine and chlorpromazine in damaging and killing worms. Surprisingly, mutations in the genes responsible for these drugs’ anti-depressant or anti-psychotic effects in humans did not eliminate their anthelmintic activity in *C. elegans* and, in some cases, increased it (Supplementary Table S3). Furthermore, *C. elegans* mutants resistant to existing anthelmintics acting on a variety of targets (from ion channels to microtubules) retained sensitivity to all three drugs, suggesting that they act on different targets. Because some dopaminergic and serotonergic pathway mutants had moderately increased sensitivities to the drugs relative to wild-type *C. elegans*, the drugs may act via these pathways in a mechanistically distinct manner from their actions in humans. Novel pathways may potentially underlie their effects in helminths as well. The target or targets responsible may be evolutionarily conserved in nematodes and trematodes as all three drugs had anthelmintic activity vs. *T. muris, A. caninum* and *S. mansoni*, as well as *C. elegans*. Sertraline, paroxetine and chlorpromazine may, therefore, be broadly effective against a range of parasitic helminth species, increasing their utility in large population-based chemotherapy efforts. They may also represent new classes of anthelmintic drugs that could be used in combination with existing front-line drugs to boost effectiveness of anti-parasite treatment as well as forestall the development of parasite drug resistance.

## Materials and Methods

### C. elegans strains

Initial library screening was performed on *C. elegans* strain CK423 *bus-8(e2698)*; *bkIs423[Psnb-1::hTDP-43* + *myo-2::dsRED]*^49^. Re-testing was done in CB6055 *bus-8(e2698)* and in the Bristol N2 wild-type isolate. Strains DA2109 *ser-7(tm1325)*; *ser-1(ok345)*, MT9668 *mod-1(ok103)*, AQ866 *ser-4(ok512)*, MT8944 *mod-5(n822)*, MT14984 *tph-1(n4622)*, JT513 *nrf-5(sa513)*, JT525 *nrf-6(sa525)*, CB3474 *ben-1(e1880)*, CB193 *unc-29(e193)*, CB306 *unc-50(e306)*, DA1316 *avr-14*(*ad1305*); *avr-15*(*vu227*) *glc-1*(*pk54*), NM1968 *slo-1*(*js379*), RB2119 *acr-23*(*ok2804*), HY496 *bre-1*(*ye4*), LX704 *dop-2(vs105); dop-3(vs106)*, OH313 *ser-2(pk1357)*, CX12800 *ser-3(ad1774)*, RB1631 *ser-3(ok2007)*, KP4 *glr-1(n2461)*, MT13113 *tdc-1(n3419)*, MT9455 *tbh-1(n3247)*, RM2702 *dat-1(ok157)*, and CB1112 *cat-2(e1112)* were utilized for genetic analysis of anthelmintic drug mechanism of action. Some strains were provided by the *Caenorhabditis* Genetics Center (CGC) funded by NIH Office of Research Infrastructure Programs (P40 OD010440), and the National Bioresource Project (Japan). WormBase (release WS251) was utilized for *C. elegans* genetic information.

### Primary anthelmintic screening against *C. elegans*

Two hundred eighty-one compounds from the NIH Clinical Collection library (2013) were screened semi-quantitatively for effects on *C. elegans* growth and viability. To conduct the screen, 2 mL of nematode growth medium (NGM) were added into 12-well cell culture dishes. Each well was seeded with 20 µL of 10x concentrated OP-50 *E.coli*. After the bacterial lawn dried, bacteria were killed by UV irradiation to minimize metabolism of test compounds by live bacteria. Serial dilutions in DMSO of each drug in 20 µL total volumes were added to individual wells, to 50 µM, 25 µM, or 12.5 µM final concentrations. Twelve to 18 eggs of *bus-8(e2698)*; *bkIs423[Psnb-1::hTDP-43* + *myo-2::dsRED]* were plated and scored for survival, viability, and progeny production after 4 and 8 days (data summarized in Supplementary Table S1). The *bus-8(e2692)* mutation was included in the initial screening due to its greater cuticle permeability to small molecules^50^. Compounds that caused lethality or growth inhibition were re-tested against *bus-8(e2698)* at 50 µM final concentration in 2 mL NGM. Subsequent side-by-side testing of *bus-8(e2692)* and N2 indicated the two strains had similar sensitivities to sertraline, paroxetine, and chlorpromazine (sertraline (#047897) and paroxetine (#047895) (Matrix Scientific, Colombia, SC); chlorpromazine (sc-202537, Santa Cruz Biotechnology, Santa Cruz, CA). Therefore, all following experiments utilized N2 (wild-type) *C. elegans*. For expanded dose-range testing, the indicated concentrations of drug were added to 2 mL NGM in 12-well dishes.

### Severity score assignments for *C. elegans*

*C*. *elegans* were developmentally stage-matched by sodium hypochlorite treatment to extract embryos^51^. Fifty to 100 N2 or mutant *C. elegans* at embryo, L1, L3/4, or gravid adult stages were seeded onto the plates in duplicate wells at each concentration of drug tested, and scored at 24, 48, and 72 h with additional plate surveys at 5 d to identify any delayed population growth. Scoring “points” were assigned at 48 h after exposure, and based on phenotypes observed throughout the total population in each well. Points were assigned for each phenotype observed, resulting in a final severity score for the drug dose tested (Table 1). Phenotypes scored were developmental delay (at least 1 developmental stage delayed compared to control, 1 point); developmental arrest (at L1 or L2 stages, 1 point); lethargy (lack of spontaneous crawling, 1 point); paralysis (lack of crawling even when stimulated but with head movements or nose retraction following stimulation, or observable pharyngeal pumping, 1 point); toxic (>90% lethality, 2 points); or death (100% lethality, 4 points). Movement was stimulated by gently touching affected animals with a thin platinum wire. If an animal failed to respond to gentle touch and had no observable pharyngeal pumping, it was scored as dead. Each drug concentration was tested in duplicate within an experiment, and in three independent experimental replicates. Adult worms were not scored for developmental delay or arrest, as they are fully developed. This decreased the number of points available to them, so may have led to an underrepresentation of drug toxicity in adults relative to younger developmental stages.

### *C. elegans* embryo lethality scoring

Fifty to 100 *C. elegans* embryos were placed onto NGM plates containing the indicated concentrations of drug. Individual hatched embryos were quantitatively scored at 48 h for survival. Paralysis or lethargy was distinguished from lethality by gently touching affected animals with a thin platinum wire and surveying pharyngeal pumping. If an animal failed to respond to gentle touch and had no visible pharyngeal pumping, it was scored as dead. Each drug concentration was tested in triplicate and results averaged.

### WormLab *C. elegans* video tracking

Stage matched N2 worms were grown at 20 °C to day 1 of adulthood. Approximately 50 day 1 adults were transferred onto NGM plates with drugs at the indicated concentrations for 18 hours. Using the video capture and analysis features of the software WormLab (Version 4.1, MBF Bioscience, Williston, VT, USA), 60 s video recordings with a resolution of 2592 × 1944 pixels were taken at 14 frames/s using a Basler acA2500 digital camera. Worms with tracking times greater than 30 s were used in the final analysis. Straight-line speed was calculated from the positional change starting at the beginning of track to end of track (µm) and then divided by track recording duration (s). Each drug concentration was tested in triplicate and results averaged.

### Anthelmintic resistance testing of *C. elegans* mutant strains

N2 and mutant *C. elegans* embryos were tested in parallel for resistance to sertraline, paroxetine, and chlorpromazine. Embryos were seeded onto 12-well plates in duplicate for each drug concentration (25 μM, 50 μM, 100 μM, or 150 μM). N2 and mutant worms were also monitored on DMSO-only control plates to account for any developmental or movement abnormalities resulting from genetic mutations. Each population was observed on multiple successive days. The qualitative observations of rates of growth and survival were recorded as described above for severity score assignment, and overall differences in drug sensitivities for mutant *C. elegans* relative to N2 were summarized in Supplementary Table S3.

### Electropharyngeogram recordings

Devices (‘chips’) were fabricated using soft lithographic methods^52,53^. Each chip had eight recording modules, as described previously^16,33^. Worms (N2 day 1 adults) were positioned (‘loaded’) via a network of channels into recording modules in the chip. Electrical signals were led to differential amplifiers and the amplified, filtered signals were digitized and acquired in Spike2 software (2500 kHz/channel; Cambridge Electronic Design; Cambridge, England). Before loading, worms were pre-incubated in M9 buffer^54^ containing 10 mM 5HT (Sigma H7752; St. Louis, MO), henceforth termed ‘M9-5HT,’ to stimulate pharyngeal pumping^17^. M9-5HT was used for loading and then perfused via a syringe pump for a 30 min baseline EPG recording period. The perfused solution was then switched to M9-5HT containing a drug or solvent and EPG recording continued for an additional 60 min. Drug solutions in M9-5HT were prepared by serial dilution of stock solutions, with the highest concentration of solvent (DMSO) being 0.05%; this concentration was used for control recordings.

Spike2 data were down-sampled to 500 Hz/channel, exported to Igor Pro (WaveMetrics, Lake Oswego, OR, USA) and analyzed using a pump-recognition algorithm^16^. Worms randomly lodged head- or tail-first in recording modules; for analysis, recordings from tail-first worms were flipped to direct the ‘E’ (muscle excitation) spikes upward^55^. The software identified the ‘E’ and ‘R’ (muscle relaxation) spike of each pump. To calculate pump frequency vs. time for each worm, we binned the time axis (1 s bin width), counted the pumps in each bin, and smoothed the result using a Gaussian weighted sliding window (S.D. = 10 s). The result was averaged across worms in each experimental group (ensemble average; *N* = number of worms in the ensemble) and plotted as mean (line) ± 1 S.E.M. (shading). Pump frequency was normalized [i.e., divided by the mean pump frequency during a 10 min period (*t* = −12 min to −2 min) near the end of the baseline period] before averaging across worms^16^. To derive IC_50_ values (concentration at which pump frequency was reduced by half), we calculated the mean pump frequency (non-normalized) in a time window during which the pump frequency was at approximately steady state (*t* = 55–60 min), and averaged the result across worms in each experimental group (i.e., each drug concentration).

### Anthelmintic testing against *T. muris*

Stock concentrations of sertraline, paroxetine and chlorpromazine were prepared by dissolving 1 mg of each drug in 10–20 µL of DMSO and then topping up with 1 mL of culture media (RPMI 1640 media containing 10% fetal bovine serum and antibiotic/antimycotic (AA)) to make 1 mg/mL concentration. From this stock solution, four different concentrations (334 µg/mL, 11 µg/mL, 0.3 µg/mL and 0.01 µg/mL of each drug were prepared for testing against the helminth nematode, *Trichuris muris*. These concentrations were converted to molarity in the Results section. Mice were orally infected with approximately 200 *T. muris* eggs. After 6 wk the mice were sacrificed and adult worms harvested from the caecum. The worms were washed with PBS/2 × AA and resuspended in 200 µL culture medium. Parasites (3 worms/well) were transferred to sterile 96-well flat bottom microplates (Corning 3650, Life Sciences, USA) and incubated overnight at 37 °C with 5% CO_2_ for background assessment and quality checks. Inter-well spaces were filled with 300 µL of culture medium to prevent dessication in the wells with worms and maintained in an incubator at 37 °C with 5% CO_2_ until the end of experiment. *T. muris* were prepared in triplicate wells for each drug and concentration. Wells were treated with different drugs (100 µL volume added per well) of four different concentrations and the motility of the worms was monitored for 24 h. The effects of drugs on the motility of worms were assessed using published methods^56^. Briefly, a 5 s video recording was taken of each well of the 96 well plate using a dissection microscope (Olympus) fitted with a Nikon D200 camera after 1, 4, 8, 12 and 24 h. Each 5 s video capture of each well was processed using the software Image J (1.47 v, imagej.nih.gov/ij) and a modified macro (“wiggle index”). On each 96-well plate, test compounds, medium control and a 1% DMSO control were arrayed in triplicate. Changes in light intensity recorded during the 5 s capture were transformed into a motility index, which included a Gaussian image blur normalization on each digitized frame of each video. The 150-frame video was transformed into a projection-stack and for each pixel the standard deviation (S.D.) of light intensity was calculated for sets of 50 frames in a ‘rolling’ manner. The mean of the S.D. of each pixel from the S.D. of light intensity of each set of 50 frames was calculated to compute the final motility index for each well. Optimal thresholds were determined prior by the analysis of untreated worms (100% motility) and media only wells (0% motility). Motility data were obtained at 1, 4, 8, 12 and 24 h after adding drugs or control solutions to the wells. The James Cook University (JCU) animal ethics committee approved all experimental work involving animals (Ethic approval number A2271).

### Anthelmintic testing against *A. caninum*

The effect of sertraline, paroxetine and chlorpromazine on hookworm larval development was assayed using center well organ culture dishes (Falcon 353037). The inner well of each dish was filled with 2 mL NGM agar. After solidification, 40 μL of drug at the desired concentration was added to each plate and allowed to dry, followed by 10 μl of an overnight *E. coli* OP50 culture that was added to the center of the agar. Hookworm eggs were isolated from feces of infected dogs by salt flotation, washed, and suspended at a concentration of 10 eggs/μL in BU buffer (50 mM Na_2_HPO_4_, 22 mM KH_2_PO_4_, 70 mM NaCl)^57^. Approximately 100 eggs (10 μL) were added to each plate and the exact number counted under a dissecting microscope. Four mL of BU buffer were added to the surrounding moat, and the dishes incubated at 27 °C for 4 d at which time the plates were scored for worm development. Larvae that progressed beyond the L1 stage were considered to be developing. Any larvae that left the plate and became trapped in buffer in the outer moat were collected and counted. Treatments were performed in triplicate, and the experiment repeated twice. All animal experiments were carried out in strict accordance with the recommendations in the Guide for the Care and Use of Laboratory Animals of the National Institutes of Health and under a protocol approved by the George Washington University Medical Center Institutional Animal Care and Use Committee.

### Anthelmintic testing against *S. mansoni*

Maintenance of the *S. mansoni* life cycle, preparation of somules and adult worms, compound storage, and co-incubation of somules or adult worms with test compounds were as described^58–60^. Somules and adults were cultured in the presence of compounds over five concentrations (0.625, 1.25, 2.5, 5, and 10 µM) for up to 48 h and 24 h, respectively. As reported previously, simple descriptors were employed to describe the observable effects of compounds on parasites (changes in shape, motility and density; see legend to Table 2)^58,61^ using an inverted microscope at 18 and 48 h post-exposure (somules) or 11 and 24 h post-exposure (adults). To convert these observations into a partially quantitative output for comparing drug effects, each descriptor was awarded a score of 1 and these were summed to a maximum score of 4^62–64^. Evidence of degeneracy or death was awarded the maximum score of 4. For adults specifically, damage to the tegument (outer surface) was also awarded a score of 4 on the understanding that surface damage is lethal to the parasite *in vivo*^65^. For somules, experiments utilized 40 animals per well per treatment. For adult assays, 5 adult male worms were tested per well per treatment. All assays were performed twice, each in duplicate.

For adult *S. mansoni*, we additionally employed WormAssay to measure worm motility^64,66^. WormAssay uses a commodity digital movie camera connected to an Apple Macintosh computer that operates an open source software application to automatically process multiple wells (in 6-, 12- or 24-well plates). The application detects worm-induced changes in the occupation and vacancy of pixels between frames. Worm motion was quantified using the “Consensus Voting Luminance Difference” option. Motility assays were repeated twice, each in duplicate.

### Statistical analysis of dose-response curves

For graphical display and statistical analysis of dose-response data we calculated the mean (*m*_*i*_) and standard error of the mean (*σ*_*i*_) of the responses at each drug concentration (*x*_*i*_) by averaging across trials. In cases where the measurement involved counting individual events (e.g., the number of living worms), *σ*_*i*_ was adjusted using the Wilson continuity correction^67^.

To test for a significant effect of the drug on the measured response, we used a modification of the likelihood ratio test to determine whether the Hill equation with 4 parameters (maximum response, minimum response, IC_50_ and Hill coefficient) provided a better fit to the data than a constant, using a modification of the likelihood ratio test^68^. To compare dose-response curves between experimental treatments, Hill curves (constrained to approach zero at high drug concentrations) were fit to the data:

1 $${H}_{i}={R}_{0}(\frac{{b}^{r}}{{x}_{i}^{r}+{b}^{r}})$$

where *R*_0_ is the response without drug, *x*_*i*_ is the drug concentration, *b* = IC_50_ and *r* is the Hill coefficient. We estimated the three free parameters (*R*_0_, *b*, *r*) using the Igor Pro FuncFit operation to minimize the sum of squares, $\sum _{i}{z}_{i}^{2}$, where ${z}_{i}=({H}_{i}-{m}_{i})/{\sigma }_{i}$ is the deviation of the observed mean response from the Hill curve, normalized by dividing by *σ*_*i*_. This weighted least squares criterion yields the maximum likelihood value of IC_50_ under the approximation that the measurement errors are drawn from independent Gaussian distributions. To test for significant differences between IC_50_ values for two dose-response curves we used the likelihood ratio test^69^, which is based on the reduction in goodness-of-fit when the IC_50_ values are constrained to be the same for the two curves, which reduces the number of free parameters from 6 (three for each curve) to five. The likelihood ratio (LR) test statistic is:

2 $$-2\,\mathrm{ln}(LR)=\sum _{i}({z}_{i}^{2}-{z}_{i}^{^{\prime} 2})+\sum _{j}({z}_{j}^{2}-{z}_{j}^{^{\prime} 2})$$

where the subscripts *i* and *j* denote the points on the two dose-response curves, ${z^{\prime} }_{i}=({H^{\prime} }_{i}-{m}_{i})/{\sigma }_{i}$ and ${z^{\prime} }_{j}=({H^{\prime} }_{j}-{m}_{j})/{\sigma }_{j}$ are the normalized deviations of the data from the constrained Hill curves ${H^{\prime} }_{i}$ and ${H^{\prime} }_{j}$. The test statistic is distributed approximately as *χ*^2^ with 1 degree of freedom, from which we determined *p*-values.

## Electronic supplementary material

Supplementary Information
