# Supplementary material for: Sertraline, Paroxetine, and Chlorpromazine Are Rapidly Acting Anthelmintic Drugs Capable of Clinical Repurposing
Source: Sci Rep. 2018 Jan 17;8:975. doi: 10.1038/s41598-017-18457-w (PMC5772060; doi:10.1038/s41598-017-18457-w)
Supplement: Supplementary file 1 — Supplementary Information [file 41598_2017_18457_MOESM1_ESM.pdf]

Title: Sertraline, Paroxetine, and Chlorpromazine Are Rapidly Acting Anthelmintic Drugs Capable of Clinical Repurposing.

Authors: Janis C. Weeks[1], William M. Roberts[1], Caitlyn Leasure[2], Brian M. Suzuki[3], Kristin J. Robinson[1], Heather Currey[4], Phurpa Wangchuk[5], Ramon M. Eichenberger[5], Aleen D. Saxton[4], Thomas D. Bird[4,6,7,8], Brian C. Kraemer[4,7,9,10], Alex Loukas[5], John M. Hawdon[2], Conor R. Caffrey[3], and Nicole F. Liachko[4,9]*

Supplemental Information:

Supplementary Table S1. Drugs with anthelmintic bioactivity in *C. elegans*

Compound	Clinical Use	Administration	Mechanism
Chlorpromazine	anti-psychotic	oral, injection, intravenous	dopamine receptor antagonist
Demeclocycline	anti-biotic	oral	tetracycline family member; inhibits protein synthesis
Econazole Nitrate	anti-fungal	topical, suppository	imidazole class molecule; inhibits synthesis of ergosterol thereby disrupting fungal cell membranes
Flecainide	anti-arrhythmia	oral	sodium channel blocker
Floxuridine	anti-cancer	intra-arterial infusion	pyrimidine analog; inhibits the S-phase of cell division
Fludarabine	anti-cancer	intravenous, oral	purine analog; inhibits DNA synthesis
5-Fluorouracil	anti-cancer	intravenous, topical	pyrimidine analog; irreversibly inhibits thymidylate synthase
Hexachlorophene	anti-bacterial	topical	inhibits bacterial electron transport chain of gram-positive bacteria
Miconazole Nitrate	anti-fungal, anti-parasite	topical, oral	imidazole class molecule; inhibits synthesis of ergosterol thereby disrupting fungal cell membranes
Minocycline	anti-biotic	oral, injection, intravenous	tetracycline family member; inhibits protein synthesis
Paroxetine	anti-depressant	oral	selective serotonin reuptake inhibitor
Sertraline	anti-depressant	oral	selective serotonin reuptake inhibitor
Triclosan	anti-bacterial	topical	inhibits bacterial fatty acid synthesis

Supplementary Table S2. *C. elegans* developmental stages 48 h after drug exposure

[Drug], µM	Sertraline	Paroxetine	Chlorpromazine
0 (DMSO control)	L4	L4	L4
10	L2	L2	L2/L3
25	L1/L2	L2	L2
50	L1/L2	L1/L2	L2
75	L1	L1	L1/L2
100	L1	L1	L1/L2
125	L1	L1	L1
150	L1	L1	L1

Developmental stage(s) of the entire tested *C. elegans* population (live and dead) were assessed 48 h after being plated as embryos on agar that contained sertraline, paroxetine or chlorpromazine at the indicated concentrations (see Figure 1A). "L" denotes larval stage. *N* > 80 worms per group.

Supplementary Table S3. Candidate anthelmintic pathway genes tested.

C. elegans gene	Mutation tested	Human homolog	Function/Resistance	SRT effect	PXT effect	CPZ effect
**Anthelmintic Resistance Genes**						
acr-23	ok2804	CHRNA6	monepantel resistant	N	N	N
avr-14; avr-15; glc-1	ad1305; vu227; pk54	GLRA2/3	ivermectin resistant	N	N	N
ben-1	e1880	TUBB4B	benzimidazole resistant	N	N	N
bre-1	ye4	GMDS	BT toxin resistant	N	N	N
slo-1	js379	KCNMA1	emodepside resistant	N	S	N
unc-29	e193	CHRNA4	levamisole resistant	N	N	N
unc-50	e306	UNC50	levamisole resistant	N	S	S
**Dopamine Signaling**						
cat-2	e1112	TH	dopamine biosynthesis	S	S	S
dat-1	ok157	DAT	dopamine transporter	N	N	N
dop-2; dop-3	vs105; vs106	DRD2	dopamine D2 receptor	N	N	N
**Octopamine Signaling**						
ser-3	ok2007 ad1774	ADRA1A	octopamine receptor	N	N	N
tbh-1	n3247	DBH	tyramine beta-hydroxylase	N	N	N
**Serotonin Signaling**						
glr-1	n2641	GRIA1	AMPA receptor	N	N	N
nrf-5	sa513	-	fluoxetine resistant	N	N	N
nrf-6	sa525	-	fluoxetine resistant	N	S	N
mod-1	ok103	GABRB3	serotonin gated chloride channel	N	N	N
mod-5	n822	SCL6A4	serotonin transporter	S	S	N
ser-4	ok512	HTR1D2	G-protein coupled receptors	N	N	N
ser-7; ser-1	tm1325; ok345	HTR7; HTR2	G-protein coupled receptor	N	N	N
tph-1	n4622	TPH2	serotonin biosynthesis	N	N	N
**Tyramine Signaling**						
ser-2	pk1357	HTR1A	tyramine receptor	N	N	N
tdc-1	n3419	DDC	tyrosine decarboxylase	N	N	N

Effect of drug treatment on mutant worms relative to wild-type *C. elegans*. Each drug was tested at 25 µM, 50 µM, 100 µM and 150 µM in duplicate wells, and each experiment was repeated at least twice. *N* = 50-100 worms/ well. Mutant worm development, motility and survival were compared to that of wild-type worms at the same concentrations of drug. SRT, sertraline; PXT, paroxetine; CPZ, chlorpromazine. S, sensitive (increased mutant lethality relative to wild-type); N, no difference between

wild-type and mutant responses; R, resistance (decreased mutant lethality relative to wild-type).
